# When leukemia harms the kidneys: a scoping review of lysozyme-induced nephropathy in chronic myeloid leukemia and chronic myelomonocytic leukemia

**DOI:** 10.1097/MS9.0000000000003717

**Published:** 2025-08-12

**Authors:** Eric Wah Sanji, Juste Niba, Kareen Esenwoh Azemafac

**Affiliations:** a Internal medicine department, Magnolia Regional Health Center, Corinth, Mississippi, USA; b General Medicine department, Swiss Care Hospital, Limbe, Cameroon; c Oncology service, Bamenda Regional Hospital, Bamenda, Cameroon

**Keywords:** lysozyme-induced nephropathy, chronic myelomonocytic leukemia (CMML), chronic myeloid leukemia (CML), proximal tubular injury, renal complications in hematologic malignancies

## Abstract

Lysozyme-induced nephropathy (LyN) is a rare and underdiagnosed renal complication associated with hematologic malignancies, particularly chronic myelomonocytic leukemia (CMML), and less commonly, chronic myeloid leukemia (CML). This scoping review aims to consolidate current knowledge of the pathophysiology, diagnosis, clinical features, management, and epidemiology of LyN in these patient populations.

**Methods:**

In accordance with PRISMA-ScR guidelines, a comprehensive literature search was conducted through PubMed, Embase, and Scopus from inception to March 2025. Eligible studies included adult patients diagnosed with LyN in the setting of CMML or CML, confirmed either clinically or by renal biopsy. Extracted data included study design, lysozyme levels, hematologic and renal presentations, diagnostic modalities, treatment strategies, and outcomes.

**Results:**

A total of ten studies met the inclusion criteria: five case reports, three retrospective cohort studies, and two pathology-based reviews. The majority involved CMML patients with markedly elevated serum or urinary lysozyme levels. Renal biopsies consistently demonstrated proximal tubular injury, eosinophilic cytoplasmic granules, and positive lysozyme immunostaining, with occasional leukemic infiltration. Treatment focused on the underlying hematologic malignancy, using agents such as hypomethylating agents, hydroxyurea, or tyrosine kinase inhibitors. Renal outcomes varied, with four patients progressing to end-stage kidney disease and three requiring dialysis.

**Conclusion:**

Lysozyme-induced nephropathy is a clinically significant but frequently overlooked tubulointerstitial complication in patients with CMML and CML. Early recognition facilitated by lysozyme quantification and kidney biopsy may allow for timely therapeutic intervention and improved renal outcomes. Increased clinical awareness and further prospective studies are warranted to guide screening strategies and optimize management protocols.

## Introduction

This scoping review is consistent with the TITAN Guidelines 2025 for ethical and transparent reporting, which includes the appropriate declaration of AI-assisted tools used in manuscript preparation^[1]^. Chronic myeloid leukemia (CML) and chronic myelomonocytic leukemia (CMML) are hematologic malignancies characterized by myeloid proliferation and dysregulation. CML is classified as a myeloproliferative neoplasm, predominantly driven by the Philadelphia chromosome, which results in the formation of the BCR-ABL1 fusion gene. In contrast, CMML is a myelodysplastic/myeloproliferative overlap neoplasm, distinguished by a persistent elevation in peripheral blood monocytes^[2,3]^.

**HIGHLIGHTS**

- Lysozyme-induced nephropathy (LyN) is a rare but underrecognized cause of renal injury in patients with chronic myelomonocytic leukemia (CMML) and, less frequently, chronic myeloid leukemia (CML).
- Elevated serum or urinary lysozyme levels, although inconsistently measured, are a common biochemical marker in affected patients.
- Definitive diagnosis relies on renal biopsy showing proximal tubular injury and lysozyme-positive eosinophilic granules.
- Early treatment of the underlying leukemia – often with hypomethylating agents or tyrosine kinase inhibitors – can stabilize or partially reverse renal dysfunction.
- Delayed diagnosis or leukemic infiltration of the kidney portends worse outcomes, including progression to end-stage renal disease.
- This scoping review synthesizes clinical, pathological, and therapeutic insights from 10 published studies over 15 years.

Monocytes serve as a major source of lysozyme; thus, lysozyme overproduction and its associated renal complications are more commonly seen in CMML than in CML, where the primary issue is granulocytic expansion^[4]^.

Lysozyme is freely filtered by the glomeruli and is normally reabsorbed in large quantities by proximal tubular epithelial cells^[5,6]^. Under physiological conditions, minimal lysozyme is excreted in urine due to effective tubular reabsorption^[5]^. However, in disease states marked by excessive monocyte proliferation such as CMML, serum lysozyme levels may increase significantly^[4,5,7]^. When the capacity for reabsorption is overwhelmed by sustained lysozyme overproduction, nephropathy may develop, signaling either a high disease burden or the presence of specific risk factors^[5,7]^.

Lysozyme-induced nephropathy (LyN) is a rare renal condition caused by excessive lysozyme accumulation in the kidneys, predominantly affecting the proximal tubules^[8–11]^. Clinically, it remains frequently underrecognized^[12]^. The toxic buildup of lysozyme can damage renal tubules, leading to acute kidney injury (AKI) or progression to chronic kidney disease (CKD)^[8]^. Early recognition of LyN is crucial to initiate timely and targeted therapeutic interventions.

This scoping review aims to provide a comprehensive overview of LyN in patients with CML and CMML. Specifically, we assess its prevalence, underlying pathophysiological mechanisms, diagnostic modalities, treatment strategies, and reported outcomes in this patient population.

## Methods

### Protocol and registration

This scoping review adhered to the PRISMA-ScR (Preferred Reporting Items for Systematic Reviews and Meta-Analyses extension for Scoping Reviews) guidelines. Although the protocol was not registered in a public repository, the methodology followed the JBI Manual for Evidence Synthesis.

### Eligibility criteria

#### Inclusion criteria

- Studies involving adult patients (≥18 years) diagnosed with CML or CMML
- Biopsy-proven or clinically diagnosed LyN
- Observational studies, case reports (*n* = 1), cohort studies, and case series with ≥2 patients

#### Exclusion criteria

- Animal models and in vitro studies
- Reviews, editorials, or letters lacking primary patient data

### Information sources

Database searches were conducted in:

- PubMed
- Embase
- Scopus

#### Search period

From database inception to March 2025

### Search strategy

Search terms combined Medical Subject Headings (MeSH) and keywords as follows:

(“Lysozyme” OR “Muramidase”) AND (“Nephropathy” OR “Tubular Injury” OR “kidney disease”) AND (“CML” OR “Chronic Myeloid Leukemia” OR “CMML” OR “Chronic Myelomonocytic Leukemia”)

### Selection process and screening process

**Figure 1.**
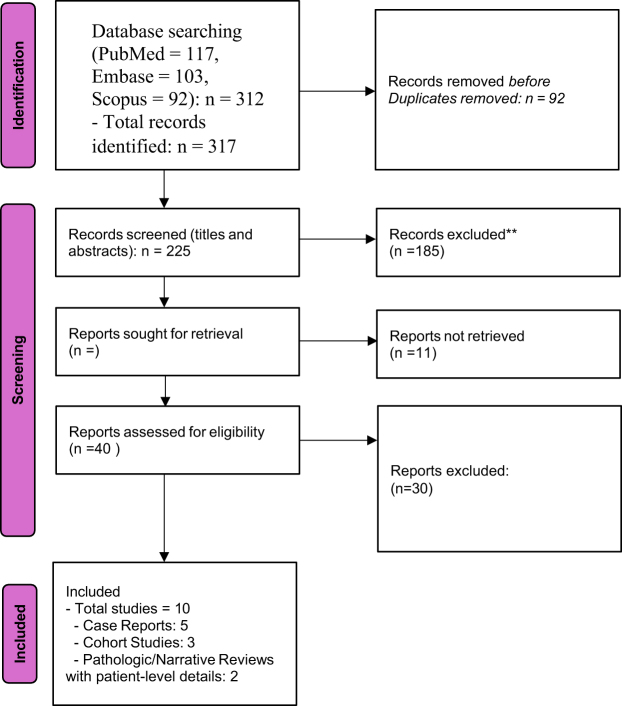
PRISMA-ScR flow diagram illustrating the selection process of studies included in the scoping review. PRISMA-ScR, Preferred Reporting Items for Systematic Reviews and Meta-Analyses extension for Scoping Reviews.

Two independent reviewers screened titles and abstracts using the Rayyan platform. Full texts of potentially relevant studies were reviewed to confirm eligibility. Discrepancies were resolved through discussion and consensus (Fig. 1).

### Data charting

A standardized data extraction form was used to capture the following:

- Study design
- Number of patients
- Type of leukemia (CML or CMML)
- Renal presentation
- Diagnostic modalities
- Serum and/or urinary lysozyme levels
- Renal biopsy findings
- Treatment approaches and outcomes

## Results

### Summary of included studies

Ten studies met the inclusion criteria for this review. These included five case reports, three retrospective cohort studies, and two narrative or pathological reviews. The studies span approximately 15 years, underscoring the rarity of LyN in hematologic practice.

Most of the reviewed literature focused on CMML, likely due to its stronger association with monocytosis and lysozyme overproduction. However, one study documented LyN in a patient with CML, indicating that this complication, although uncommon, may occur across a broader spectrum of myeloid neoplasms.

### Patient demographics and clinical presentation

Most patients were male, with an age range of 54–78 years. In most cases, a diagnosis of CMML had already been established, characterized by persistent monocytosis. The most common clinical presentations were moderate proteinuria and elevated serum creatinine, reflecting progressive renal insufficiency consistent with either CKD or AKI. Notably, in three studies, renal dysfunction was the initial presenting feature that prompted hematologic evaluation, ultimately leading to the diagnosis of CMML.

### Lysozyme measurement and diagnostic workup

Eight out of 10 studies reported serum or urinary lysozyme levels, all of which were markedly elevated and exceeded normal reference ranges. However, lysozyme quantification was not standardized across studies and varied in methodology and frequency. Renal biopsy served as the definitive diagnostic tool in most cases, as imaging findings were often non-specific or unremarkable. Histologic examination of biopsy specimens consistently revealed features of tubulointerstitial nephritis, including proximal tubular vacuolization and abundant eosinophilic cytoplasmic granules. Lysozyme presence within these granules was confirmed by immunohistochemical staining. Table 1 shows a summary of the selected studies reporting LyN in CML and CMML.

**Table 1:** Summary of Studies Reporting LyN in CMML and CML Patients

Study	Design	Leukemia Type	Lysozyme Levels Reported	Renal Findings	Treatment and Outcome
Santoriello *et al* (2016)^[5]^	Review + Pathology Cases	CMML	Not quantified	Proximal tubular injury; eosinophilic granules	Supportive care discussed
Patel *et al* (2019)^[4]^	Case Report	CMML	Plasma: 26.3 μg/mL	Protein resorption droplets; positive lysozyme IHC in tubular epithelial cells	Azacitidine; renal function stabilized post-treatment
Lafargue *et al* (2023)^[13]^	Cohort Study	CMML	Elevated (not detailed)	Interstitial infiltration; LyN features	Azacitidine, hydroxyurea, or chemotherapy; dialysis in severe cases; poor prognosis
Asano *et al* (2021)^[9]^	Case Report	CMML	Not reported	Intraglomerular infiltration; LyN	Supportive; fatal outcome
Ferreira *et al* (2024)^[14]^	Case Report	CMML	Serum: 120 mg/L; Urine: 200 mg/L	CKD with biopsy- confirmed LyN	No renal recovery; progressed to ESRD
Pal *et al* (2023)^[15]^	Case Report	CML	Elevated	Improved renal function with TKI	Dasatinib improved renal function
Kudose *et al* (2023)^[5]^	Clinicopathologic Review	CMML	Yes (quantified in some cases)	LyN histopathology spectrum	Not reported
Mohamadou *et al* (2021)^[16]^	Case Report	CMML	Plasma: 120 mg/L; Urine: 200 mg/L	Fanconi-like symptoms; biopsy-confirmed LyN	Supportive care; outcome unknown
Donati and Shirali (2021)^[17]^	Case Report	CMML	Elevated	Biopsy-confirmed tubular injury	Supportive + leukemia treatment
Robinet-Zimmermann *et al* (2020)^[10]^	Case Report	CMML	Elevated (reported)	Eosinophilic granules; positive lysozyme staining in proximal tubules.	Supportive care + azacitidine; partial renal recovery; persistent insufficiency

### Histopathologic features

The histopathological hallmark of LyN was injury to proximal tubular epithelial cells, demonstrated by cytoplasmic vacuolization, lysozyme-positive eosinophilic granules, and varying degrees of interstitial edema or fibrosis. Three cases additionally exhibited leukemic infiltration of the kidney parenchyma, suggesting a dual mechanism of renal injury in certain patients. Glomerular abnormalities and immune complex deposition were not observed, reinforcing the primarily tubulointerstitial and non-glomerular nature of LyN.

### Treatment modalities and response

Management primarily targeted the underlying hematologic malignancy. Six patients received hypomethylating agents (azacitidine or decitabine), three were treated with hydroxyurea, and one patient underwent intensive chemotherapy. Reduction in peripheral monocyte counts following treatment was associated with stabilization or improvement in renal function in several cases, supporting the hypothesized causal role of lysozyme in the pathogenesis of nephropathy. Despite therapeutic intervention, four patients progressed to end-stage renal disease, and three required renal replacement therapy.

### Prognosis and outcomes

Clinical outcomes varied. Of the 10 cases, four patients demonstrated partial recovery of renal function, two became dialysis-dependent, one died from complications related to both renal failure and leukemia, and three experienced persistently impaired but stable renal function.

Poorer outcomes were observed in patients with delayed diagnosis or leukemic infiltration of the kidney. Overall, renal damage in LyN is frequently irreversible, underscoring the critical importance of early detection and appropriate treatment.

## Discussion

This scoping review highlights the often underrecognized role of LyN in CMML, and to a lesser extent, CML. LyN arises due to excessive lysozyme production by malignant monocytes, resulting in cytotoxic accumulation within the proximal tubules. This process leads to tubulointerstitial injury, as the glomeruli freely filter lysozyme, and proximal tubular cells reabsorb it^[18]^.

Histopathologically, LyN is marked by vacuolated proximal tubules and interstitial inflammation, with confirmation via lysozyme immunohistochemical staining^[19]^. Studies have indicated that renal involvement occurs in up to 35% of CMML patients^[20]^. While the true prevalence of LyN remains undefined, it likely constitutes a substantial portion of renal complications in these patients^[8,12]^. Notably, patients with the proliferative subtype of CMML – characterized by elevated white blood cell and monocyte counts – appear to be at greater risk due to higher lysozyme production^[13]^.

A consistent theme across studies is diagnostic delay, often attributable to the non-specific nature of renal symptoms. Progressive renal dysfunction is frequently misattributed to chronic kidney disease of unrelated origin or drug-induced nephropathy, until biopsy reveals LyN as the culprit. This underscores the importance of considering LyN in patients with unexplained renal decline and monocytosis^[14]^. Although elevated serum and urinary lysozyme levels are informative, they are not routinely assessed, revealing a critical gap in current diagnostic practice.

Most included studies reported renal biopsy findings of tubulointerstitial nephritis, with abundant eosinophilic cytoplasmic droplets staining positively for lysozyme. In several cases, leukemic infiltration of the renal parenchyma was also noted, suggesting a dual mechanism of injury^[9,13]^. The absence of glomerular immune complex deposition supports the non-glomerular pathogenesis of LyN.

Therapeutically, management centers on treating the underlying hematologic malignancy to reduce lysozyme production. In CML, this typically involves tyrosine kinase inhibitors (TKIs). One case reported improvement in renal function with Dasatinib, supporting the role of TKIs in reversing LyN in CML^[15,21–23]^. In CMML, agents such as azacitidine, decitabine, and hydroxyurea were commonly used, with varying degrees of renal recovery. Early intervention was often associated with renal stabilization or partial improvement, while delayed treatment or concomitant leukemic infiltration led to progression to end-stage kidney disease requiring dialysis^[20]^.

Prognostically, LyN confers a poorer outcome. Renal dysfunction limits chemotherapeutic options and is associated with increased morbidity and mortality^[24]^. This aligns with broader evidence linking kidney impairment in hematologic malignancies to reduced overall survival^[25]^.

In summary, this review reveals that LyN remains underdiagnosed and underreported. Greater clinical awareness, consistent screening of high-risk patients, and the routine measurement of serum and urinary lysozyme levels are essential. Future research should focus on prospective studies to better define the incidence, risk factors, and optimal management strategies.

Collaborative care between nephrologists and hematologists is paramount in improving diagnosis and outcomes.

## Conclusion and future directions

Lysozyme-induced nephropathy is a rare but clinically significant complication that primarily affects patients with CMML and, less commonly, those with CML. Given its underrecognized nature, clinicians should maintain a high index of suspicion, particularly in patients presenting with acute kidney injury, proteinuria, and monocytosis. Definitive diagnosis requires renal biopsy, which typically reveals proximal tubular abnormalities and lysozyme-positive inclusions.

The occurrence of LyN often correlates with more aggressive disease variants, particularly proliferative CMML. Prompt and targeted treatment of the underlying hematologic malignancy may lead to stabilization or partial renal recovery. However, the overall prognosis remains guarded, especially in patients with delayed diagnosis or concomitant leukemic infiltration.

Future research priorities should include large-scale epidemiological studies to determine the true incidence and prevalence of LyN in CML and CMML. Prospective trials are needed to clarify specific risk factors and to evaluate standardized diagnostic thresholds for serum and urinary lysozyme levels. In addition, a deeper understanding of the molecular pathways involved in lysozyme-induced tubular damage is essential. Clinical trials testing targeted therapies for LyN, and the development of predictive biomarkers for treatment response, could significantly improve patient outcomes. Importantly, exploring the combined impact of lysozyme toxicity and direct leukemic infiltration may refine current treatment paradigms. Multidisciplinary collaboration remains essential for the timely recognition and effective management of this often-overlooked complication.

## Data Availability

Not applicable.
